# The current application of 3D printing simulator in surgical training

**DOI:** 10.3389/fmed.2024.1443024

**Published:** 2024-08-29

**Authors:** Yang Jiang, Hanyu Jiang, Zhikun Yang, Ying Li

**Affiliations:** ^1^Department of Ophthalmology, Peking Union Medical College Hospital, Chinese Academy of Medical Sciences, Beijing, China; ^2^Key Laboratory of Ocular Fundus Diseases, Chinese Academy of Medical Sciences & Peking Union Medical College, Beijing, China; ^3^Eight-Year Medical Doctor Program, Chinese Academy of Medical Sciences & Peking Union Medical College, Beijing, China

**Keywords:** 3D printed, surgical training, medical education, simulator, advances

## Abstract

In the rapidly evolving field of medical education, the integration of innovative technologies has become paramount to enhance the training and proficiency of future surgeons. Among these advancements, the application of 3D printing technology stands out as a useful tool in surgical training. The advantages of the 3D printing model include customization, re-usability and low-cost. The average cost of the 3D printing simulators was between $100–1000. However, there were extremely high potential labor cost during the 3D printing that hadn’t been calculated into. Additionally, in the current stage, the 3D printing simulator still have specific limitations. The most mentioned limitation was poor haptic feedback of the simulators, which was very important during the surgical training, since it is the key element for junior doctors to master practical procedures. Also, some simulators didn’t possess the integrated and elaborate structure as the human tissue, hence not the whole surgical procedures can be practiced by the trainees, and further improvement should be made. Although there are shortages, many studies have proved that 3D printing simulator can effectively reduce learning curves and is useful to enhance the trainees’ surgical skills.

## 1 Introduction

Three-dimensional (3D) printing technology has revolutionized our perception of how advanced technologies contribute to medical education and clinical practice. Patient-specific or personalized 3D-printed models have been increasingly used for medical applications, with research findings proving its value in different aspects. Seifert et al. (1), Nica et al. (2) and Sonkaya and Bek Kürklü (3) have conduct different 3D-printed models for oral and maxillary surgery course, Alrasheed et al. (4) and Suzuki et al. (5) developed 3D-printed models for endoscopic sinus surgery training, Barber et al. (6), Chien et al. (7), Frithioff et al. (8), Iannella et al. (9), Nguyen et al. (10) and Takahashi et al. (11) introduced various 3D-printed ear surgery simulator or 3D-printed temporal bone models, and the evaluations conducted by the studies all indicated that the 3D models were useful training/educational tool. Narayanan et al. (12) evaluated the efficacy of using 3D-printed models for endoscopic skull base surgery training, Famery et al. (13) and Vatankhah et al. (14) assessed the effectiveness of 3D-printed models for teaching ophthalmic surgeries, Nebor et al. (15), Uhl et al. (16), Zhu et al. (17), Zheng et al. (18), Encarnacion et al. (19) and Byvaltsev et al. (20) developed 3D-printed models for neurosurgery, and the studies all demonstrated the model’s effectiveness in enhancing surgical skills. Papavasiliou et al. (21) and Neijhoft et al. (22) evaluated the use of 3D-printed models for teaching orthopedic surgery, Campi et al. (23), Wong et al. (24), Reilly Scott et al. (25) and Hermans et al. (26) conducted studies of 3D-printed model for the urinary surgery training, Alshomer et al. (27) explored the use of desktop 3D printing to create an affordable training tool for microvascular anastomosis, and the approaches all represented their cost-effectiveness and helpfulness for enhancing surgical education.

In this article, we present the current 3D-printed models in surgical training. Our purpose is to demonstrate the usefulness of these models in medical education and clinical applications.

## 2 Search strategy

MEDLINE, EMBASE, CINAHL, ERIC, NCBI, and Web of Science were searched from Jan 1,1900 to May 30, 2024, for articles addressing 3D-printed models in surgical training. To guide our search, the search terms of “surgical training”, “3D printed model”, “curriculum”, and “education” were used combined with Boolean operators “AND”, “OR”, and “ADJACENT”. Reviews, editorials and letters would be excluded from the research. There were 58 papers founded by the search strategy, and among them, 24 papers were excluded according to the criteria of exclusion. In the end, there were 34 studies enrolled into the review.

## 3 The current 3D printing simulators for surgical training

### 3.1 3D printing simulator for oral and maxillofacial surgery

Seifert et al. (1) compared 3D-printed patient-specific models with cadaver models in an undergraduate oral and maxillary surgery course. The 3D-printed model was made to simulate the realistic extraction of the impacted third molars. Thirty-eight dental students participated, finding that 3D-printed models were significantly more realistic in anatomical accuracy and operative simulation. The 3D-printed models were also more cost-effective. However, a less fragile silicone gingiva and a better differentiation between the color of teeth and bone were the most desired suggested improvements to the 3D-printed patient individualized models. Nica et al. (2) investigated the effectiveness of using 3D-printed polymeric maxillary models in ex vivo training for dental practitioners. The technical steps of sinus augmentation and implant insertion could be performed on the corresponding 3D-printed he mi-maxillary prior to the real in vivo surgery, which included incision, flap elevation, sinus membrane elevation, flap repositioning, and suture. Results showed that the group with ex vivo training had significantly reduced operative times by 20% and similar surgical outcomes compared to the control group, demonstrating the benefit of 3D-printed models for enhancing surgical precision and efficiency. Sonkaya and Bek Kürklü (3) investigated the educational impact of a 3D-printed tooth model versus standard plastic models and extracted teeth for pre-clinical dental training. Fifty-five students rated the 3D-printed tooth model highly for its realism and usefulness in learning cavity preparation and pulp capping. The study concluded that 3D-printed models provided a cost-effective, realistic training tool, enhancing students’ practical skills before clinical practice.

### 3.2 3D printing simulator for surgery training in otolaryngology

#### 3.2.1 3D-printed models for endoscopic sinus surgery training

Alrasheed et al. (4) developed and validated a 3D-printed model for endoscopic sinus surgery (ESS) training. The model was meant to reproduce a range of skills required for ESS, including injection of the middle turbinate; pledget insertion into the middle meatus; middle turbinate medialization and the identification of key landmarks (ethmoid bulla, maxillary ostia, frontal recess); uncinectomy; frontal recess dissection; and anterior ethmoidectomy. The study involved 20 participants, including rhinologists and otolaryngology residents, who completed 6 prespecified tasks including maxillary astronomy and frontal recess dissection on the simulator. Participants evaluated the model using survey ratings based on a 5-point Likert scale. All the participants felt that the simulator would be useful as a training/educational tool (4.6/5). The following responses were obtained: visual appearance 4.25/5; realism of materials 3.8/5; and surgical experience 3.9/5. Participants found the simulator to be a valuable training tool, suggesting its integration into rhinology training curricula. Suzuki et al. (5) evaluated the educational benefit of repetitive simulation training using 3D-printed sinus models for functional endoscopic sinus surgeries (FESS). The simulation training was started with uncinectomy and maxillary antrostomy, followed by anterior and posterior ethmoidectomy, sphenoidotomy, and frontal sinusotomy. Forty-seven otolaryngologists, categorized into experts, intermediates, and novices, performed FESS on the models. Their performance was assessed using The Objective Structured Assessment of Technical Skills (OSATS) scores and post-dissection CT scans. The study found significant differences in performance across experience levels, validating the model’s construct. Novices who underwent repetitive training showed marked improvement in OSATS scores and dissection quality, indicating that the 3D models effectively enhanced surgical skills.

#### 3.2.2 3D-printed models for otologic surgical skill training

Barber et al. (6) developed a 3D-printed pediatric endoscopic ear surgery simulator to enhance surgical training. The simulator, designed using Computer Aided Design (CAD) software and fabricated with calcium sulfate hemihydrate powder, aimed to improve technical skills for trans canal endoscopic ear surgery (TEES). Six trainees tested the simulator, showing significant improvement in task completion times, indicating construct validity. The study concluded that the 3D-printed simulator is a cost-effective, high-fidelity tool for developing essential skills for TEES, demonstrating its potential for widespread use in otolaryngology training programs. Chien et al. (7) validated a 3D-printed human temporal bone model for otologic surgical skill training. The model was made to perform a series of otologic procedures, including cortical mastoidectomy, epitympanectomy, posterior tympanotomy, cochleostomy/round window surgery, canalplasty, canal wall down mastoidectomy, labyrinthectomy, and temporal bone resection. Seventeen otolaryngology trainees completed a series of otologic procedures using the model and rated its anatomical realism and training utility. Results showed that the model was highly realistic and useful for teaching temporal bone anatomy and surgical techniques. Although, the drill tone and color contrast were felt to be less realistic than cadaveric temporal bones, participants recommended incorporating the model into training curricula, indicating it as a viable alternative to cadaveric temporal bones due to its safety, accessibility, and effectiveness in developing surgical skills.

Frithioff et al. (8) investigated the effectiveness of 3D-printed temporal bone models compared to virtual reality (VR) simulation for mastoidectomy training. Eighteen otorhinolaryngology residents trained on 3D-printed models outperformed a control group that trained on VR simulations by 29% during subsequent cadaveric dissection. Although, the present models lack realistic representation of soft tissue, such as the facial nerve, dura and sigmoid sinus, this study demonstrated that 3D-printed models could improve technical skills more effectively than VR simulations, highlighting their value as a supplementary tool for traditional cadaver training. Iannella et al. (9) introduced the ‘SAPIENS’ 3D-printed temporal bone model, developed for educational and surgical simulation purposes. This model was created through a multidisciplinary collaboration and demonstrated high anatomical accuracy and it can stimulate mastoidectomy, epitympanotomy, posterior tympanotomy, and canal wall down technique. Evaluations by experienced ears, nose, and throat (ENT) surgeons indicated that the model closely resembled human temporal bone anatomy, with particular strengths in simulating mastoidectomy and middle ear dissection. Although, the 3D-printed models may not fully replicate the sensation of real tissue or bone, the questionnaire-based assessment by five experienced ENT surgeons still yielded a relatively high average total score of 49.4 ± 1.8 out of 61. The study concluded that the ‘SAPIENS’ model is a valuable tool for otologic surgery training, offering a cost-effective and realistic alternative to cadaveric bones. Nguyen et al. (10) modified a commercially available 3D-printed temporal bone model to include force sensors for stapes fixation surgery training. This study involved six junior and seven senior surgeons performing piston prosthesis placement and crimping tasks. The results showed that senior surgeons applied significantly lower forces to the stapes compared to junior surgeons, highlighting the model’s potential as a training tool to help residents self-evaluate their progress. The modified model demonstrated high fidelity in anatomical representation, offering an effective means for teaching delicate middle ear surgeries. Takahashi et al. (11) addressed the limitations of conventional 3D-printed temporal bone models by making slight manual modifications. These modifications included creating drainage holes for removing excess materials, converting ossicles ligaments to bony structures, and differentially coloring small and soft tissue structures. The model was evaluated through macroscopic and endoscopic inspections, Computed tomography (CT) imaging, and surgical performance assessments by 20 otolaryngologists. The study found that the model was highly reproducible for most anatomical structures, though the stapes, tympanic sinus, and mastoid air cells were less satisfactory. Surgeons rated the model highly for tactile sensation and anatomical accuracy, suggesting its utility for surgical training.

#### 3.2.3 3D-printed models for skull base surgery training

Narayanan et al. (12) evaluated the efficacy of using 3D-printed models with preexisting pathology for endoscopic skull base surgery training. The study involved 15 ENT surgeons participating in a workshop where they used models created from Magnetic Resonance Imaging (MRI) and CT imaging data of a patient with platybasia and basilar invagination. The models allowed surgeons to practice complex procedures such as skull base drilling and navigation. Participants rated the models highly for their anatomical accuracy and the realism of surgical tasks, indicating that these models are valuable tools for structured, simulation-based surgical training.

### 3.3 3D printing simulator for ophthalmology surgery

Famery et al. (13) introduced a new wet lab model for Descemet membrane endothelial keratoplasty (DMEK) using an artificial anterior chamber equipped with a 3D-printed iris. This DMEK wet lab model offered a teaching technique that permit to perform all DMEK surgical steps. It also offered the possibility of varying the surgical difficulty by changing the anterior chamber depth. This model, tested by both beginners and experienced surgeons, allowed for the complete simulation of DMEK procedures, including graft preparation, insertion, and orientation. The study found that experienced surgeons performed significantly faster and with higher performance scores compared to beginners. The model proved to be a realistic, resource-sparing teaching tool, effectively replicating surgical steps and accommodating adjustable surgical difficulties. Vatankhah et al. (14) assessed the effectiveness of 3D-printed models for teaching orbital anatomy, anomalies, and fractures to ophthalmology residents. In a quasi-experimental study, 24 residents were divided into control and intervention groups, with the latter using 3D-printed models for training. Results indicated that the intervention group showed significant improvement in post test scores compared to the control group, particularly among first-year residents. This study highlighted the positive impact of 3D modeling on learning outcomes, demonstrating that 3D-printed models are effective tools for enhancing anatomical understanding and surgical skills.

### 3.4 3D printing simulator for neurosurgery

#### 3.4.1 3D printing simulator for dural repair, cranioplasty and intracerebral hematoma (ICH) evacuation surgery

Nebor et al. (15) developed a 3D-printed model for primary dural repair via an endoscopic endonasal corridor to enhance surgical training. Using an Ultimaker 2++ printer, they created a cranial base and nasal cavity replica fitted with chicken skin to simulate dura mater. Twenty-six participants, categorized by experience levels, performed suturing tasks using the model. Most participants rated the model highly for improving technical skills and endoscopic proficiency, particularly in dural suturing. Novices showed significant improvement in task completion time, demonstrating the model’s effectiveness as a cost-efficient training tool. Uhl et al. (16) conducted a study on the use of 3D-printed models for surgical simulation of cranioplasty in craniosynostosis. Ten neurosurgeons with less than five years of experience were divided into two groups: one trained traditionally with cadaveric models and the other with 3D-printed models. The study found that surgeons using 3D-printed models showed better accuracy in bone reshaping and fixation and improved overall performance compared to those trained traditionally. Participants reported that the 3D models provided a realistic and controlled environment for practicing surgical techniques, enhancing their understanding and skills. Zhu et al. (17) developed a 3D-printed model for training endoscopic and exoscopic intracerebral hematoma (ICH) evacuation surgery using a tubular retractor. The model included a skull frame and a replaceable module filled with edible gelatin and animal blood to mimic brain tissue and hematoma. Twenty neurosurgeons and five postgraduate students participated in the training, showing significant improvements in their skills over multiple sessions. The model was rated highly for its value as a training tool (3.65/4) and realism in simulating surgical procedures. The model received good comments regarding the bone texture (mean: 3.20), the brain tissue texture (mean: 3.20), and the experience in aspirating the hematoma (mean: 3.10). This study demonstrated the model’s effectiveness in enhancing surgical skills in ICH evacuation.

#### 3.4.2 3D printing simulator for endoscopic surgery for pituitary adenomas

Zheng et al. (18) assessed the practical value of multimaterial and multicolor 3D-printed models for training in transnasal endoscopic surgery for pituitary adenomas. The multimaterial and multicolor model was used to simulate the endoscopic procedures: the removal of elastic turbinate and nasal septum; the removal of the anterior wall of the sphenoid sinus and the exposure of tumor. The study included three types of 3D-printed models evaluated by 12 residents and 6 experienced neurosurgeons. Results showed that the multimaterial and multicolor model provided superior anatomical teaching, surgical training, and preoperative planning compared to monomaterial models. The multimaterial model offered realistic tactile feedback and enhanced understanding of anatomical relationships, demonstrating its effectiveness in improving surgical skills and planning.

#### 3.4.3 3D printing simulator for microvascular training in neurosurgery

Encarnacion Ramirez et al. (19) developed a 3D-printed brain model with vasculature for neurosurgical procedure visualization and training. Using patient-specific MRI data, the model was constructed from silicone to replicate brain tissue and blood vessels. Seven residents and eight senior neurosurgeons tested the model, finding it anatomically precise and helpful for training in neuroendoscopic 3D perception and navigation. This study concluded that the model provides an indispensable tool for young neurosurgeons to gain operative experience safely and effectively without exposing patients to risk. Byvaltsev et al. (20) explored the use of 3D-printed cranial models to simulate operative field depth for microvascular training in neurosurgery. The study involved creating 3D-printed skulls with openings for different surgical approaches, allowing trainees to practice deep field microsurgery on chicken thighs and rat aortas. Results demonstrated the models’ effectiveness in providing realistic depth perception, hand positioning, and instrument manipulation, enhancing microsurgical training. The study concluded that these models improve training conditions, leading to better surgical accuracy and efficiency while minimizing patient risk.

#### 3.4.4 3D printing simulator for neuroendoscopic ventricular lesion removal

Licci et al. (28) developed and validated a synthetic 3D-printed simulator for training in neuroendoscopic ventricular lesion removal. Based on patient-specific CT data, the simulator included realistic ventricular structures and a tumor model made from polyvinyl alcohol to mimic soft-consistency lesions. The 3D-printed model was produced to simulate endoscopic ultrasonic resection of the ventricular lesions using the Endoscopic Neurosurgical Pen (ENP) (Söring GmbH). The simulator was evaluated by neurosurgical trainees, who rated its anatomical realism and mechanical properties highly. The 3D-printed model could not simulate all the surgical procedures of the neuroendoscopic ventricular lesion removal, just like a frontal approach for the insertion of a ventricular drain (e.g., the skin incision, burr hole placement, and so on), albeit its elementary importance in training young neurosurgeons on a resident’s level. Even so, the results of the study still could conclude that the simulator effectively aided in developing endoscopic surgical skills, providing a valuable, low-cost, reusable training tool that could enhance procedural competence in a risk-free environment for the young neurosurgeons.

### 3.5 3D printing simulator for orthopedics surgery

Papavasiliou et al. (21) developed a standardized simulation training module using a 3D-printed flexor tendon model to improve surgical training for plastic surgery residents. The model was used to train a two-strand modified Kessler technique, four-strand cruciate technique, four-strand adelaide technique with an epitendinous running suture and Silfverskiöld epitendinous suture. Twenty-eight Senior House Officers participated in the study, which involved a step-ladder approach with increasing levels of difficulty. Because the tendon of the 3D printed simulation model have already been exposed and therefore the trainees could not practice on dissecting the overlying tissue (raising Brunner flaps, protecting neurovascular bundle, tendon retrieval and pulley exposure). Even so, the training was still considered with significantly improve of participants’ knowledge and practical skills. All trainees confirmed that the training module improved their confidence with flexor tendon repair. The study concluded that the 3D-printed model was an effective tool for enhancing surgical skills in tendon repair, providing a cost-effective, ethical, and reproducible training method. Neijhoft et al. (22) evaluated the use of 3D-printed models for teaching and surgery of transitional fractures of the ankle. Utilizing CT data, the models were printed and used in hands-on teaching courses. The study involved pediatric traumatology courses where fracture anatomy, surgical approaches, and screw placement were demonstrated and practiced. Participants found the models effective for their understanding of complicated fractures, allowing them to visualize and practice surgical techniques more accurately. The study concluded that 3D-printed models significantly enhanced the educational process in traumatology, offering a valuable tool for both teaching and surgical planning. Ferràs-Tarragó et al. (29) introduced a novel arthroscopy training program utilizing a 3D-printed simulator, aiming to make arthroscopy training more accessible and cost-effective. The 3D-printed model was produced to simulate the repair surgical procedure of tendon injuries. The study involved twenty first-year orthopedic residents from nine hospitals, who performed fourteen progressively challenging exercises designed to develop fundamental arthroscopic skills. The exercises were evaluated using the Arthroscopic Surgical Skill Evaluation Tool (ASSET), and results showed a mean ASSET score of 22 points. The program proved to be effective, affordable, and easy to implement, with participants reporting significant skill improvement and high satisfaction.

### 3.6 3D printing simulator for gastrointestinal surgery

#### 3.6.1 3D printing simulator for conventional gastrointestinal surgery

Habti et al. (30) developed and assessed a customized 3D-printed small bowel simulator for hand-sewn anastomosis (HSBA) training. This simulator, made from silicone and a 3D-printed clamp system, was tested by 16 junior residents. The majority rated it as a good or very good tool for learning HSBA, despite some suggestions for improving the silicone’s texture. The simulator provides a cost-effective and realistic alternative for acquiring essential surgical skills.

#### 3.6.2 3D printing simulator for laparoscopic surgery

Yang et al. (31) conducted a prospective cohort study to evaluate a 3D-printed model for laparoscopic pancreaticojejunostomy training. The surgical procedures can be stimulated in the study including the classic biliary anastomosis and Cattell-Warren pancreaticointestinal anastomosis. Sixteen surgeons with varying experience levels participated, showing that those with higher seniority performed better initially. Although the stimulation of the pancreatic duct was considered as not realistic enough, the stepwise training program, starting with laparoscopic biliary-enteric anastomosis, indeed improved residents’ performance. The study demonstrated that repeated training with this model enhances operative skills, providing a valuable tool for mastering complex procedures in a controlled environment. Xia et al. (32) investigated a 3D-printed model for training in laparoscopic intracorporeal intestinal anastomosis (LIIA). The study aimed to design a cost-effective, reusable model and evaluate its authenticity and validity. Fifteen surgeons were divided into expert, intermediate, and novice groups to perform LIIA using the model. The results showed significant differences in performance scores and operation times across the groups, with repeated training improving the skills of novices to the level of untrained intermediates. The five experts rated the face and content validity of the model very high, with the median being four out of five. Although the intestine used in the experiment was single-layered, and it failed to simulate the actual structure of the human intestine, which is multilayered, the model still demonstrated excellent face, content, and construct validity, suggesting its potential as an effective training tool for LIIA. Xia et al. (33) designed and evaluated a portable 3D-printed model for laparoscopic choledochojejunostomy (LCJ) training. The study assessed the model’s face, content, and construct validity using the objective structured assessment of technical skills (OSATS). The operating space score of the model was 4.83 ± 0.41 points. The impression score of bile duct was 4.33 ± 0.52. The tactile sensation score of bile duct suture was 4.00 ± 0.63 and 3.83 ± 0.41points, respectively. Fifteen surgeons of varying experience levels participated, and results indicated significant differences in OSATS scores and operation times among groups. Repeated training improved residents’ performance, indicating the model’s potential for enhancing surgical skills and reducing patient risk. The current model could not contain blood vessels or simulation of intraoperative bleeding, which will more perfectly mimic the real surgery situation. Even so, this 3D-printed LCJ model offered a cost-effective, realistic training tool for biliary surgery. Holt et al. (34) developed and evaluated a 3D-printed endoscopic ampullectomy training model to improve the technical skills, knowledge, and confidence of endoscopists. The model included a reusable silicone stomach-duodenum, an ampullary mount, and a base to stabilize the duodenum. The 3D-printed endoscopic ampullectomy training model could stimulate the core procedural steps of endoscopic ampullectomy, including passing an ERCP endoscope through the pylorus to the ampulla, identification of the ampulla, positioning the snare over the ampulla, and performing snare resection, specimen retrieval, pancreatic duct cannulation, and pancreatic stent insertion. Sixteen endoscopists tested the prototype, performing core procedural steps such as ampulla identification and pancreatic duct cannulation. The model received mean technical and visual realism scores of 3.1 and 3.2, respectively, on a 1–5 scale. Although the developers identified a limitation with using a rigid base and ampulla mount in combination with a flexible stomach-duodenum because there was potential for the 2 to separate during endoscope maneuvering, the participants reported increased confidence and knowledge after using the model, indicating its potential effectiveness for training in this high-risk procedure.

### 3.7 3D printing simulator for urinary surgery

Campi et al. (23) developed and tested the RAKT Box, the first entirely 3D-printed, perfused simulator for vascular anastomoses during robot-assisted kidney transplantation (RAKT). The trainees were able to perform both the arterial and venous anastomosis on the 3D-printed simulator, which were the key steps of the kidney transplantation surgery. This study involved a multidisciplinary team and a three-year iterative development process. The simulator includes a fixed model of the human pelvis, disposable iliac vessels, and a kidney model with a hydraulic circuit to simulate arterial and venous blood flow. Although, the 3D-printed simulator could not allow simulation of ureterovesical anastomosis, while ureterovesical anastomosis is a key step in kidney transplantation, the great evaluation was still given by the users. It was tested by an expert RAKT surgeon and four trainees, demonstrating reliability and effectiveness in training key surgical steps. The study concluded that the RAKT Box was a valuable educational tool for developing RAKT-specific skills and could serve as the foundation for a structured RAKT surgical curriculum. Wong et al. (24) detailed the evolution and use of a 3D-printed bladder model for teaching urethrovesical anastomosis (UVA) during minimally invasive radical prostatectomy. Early versions were made of latex and silicone, but the current model is produced using computer-aided design and 3D printing with a polymer that simulated human bladder tissue. The model was used within a realistic pelvic silicone torso to emulate surgical conditions. It has been shown to effectively transfer skills from virtual reality robotic simulators to actual robotic surgical systems, making it a valuable tool for training residents in a controlled, risk-free environment. Reilly Scott et al. (25) investigated the use of individualized 3D-printed models for trainee and patient education, and surgical planning in robotic partial nephrectomies (RAPN). All RAPN were performed by one of six attending surgeons using a Da Vinci surgical system^®^ (Intuitive Surgical, Sunnyvale, CA) via a transperitoneal or retroperitoneal approach. The study included 40 patients and surveyed patients and surgical teams before and after exposure to the 3D models. Results indicated significant improvements in patient understanding of kidney anatomy, their disease, the surgical procedure, and associated risks after viewing the models. Surgical team members, including residents and fellows, reported increased confidence in their surgical plans. Additionally, using the models resulted in better operative outcomes, including reduced warm ischemia time and shorter hospital stays. However, the authors pointed that because computer algorithms nowadays have not yet advanced enough to control the 3D printer based on simply identifying the target lesion on both CT and MRI imaging, the interpretation of the imaging and subsequent segmentation required the cooperation of radiologist and trained printing technician, which indicated high cost of human labor and would be an essential limitation of the 3D-printed model. Hermans et al. (26) validated a 3D-printed silicone renal tumor model for RAPN training. Surgical systems used were the Da Vinci Si, X and Xi (Intuitive Surgery) with training instruments and a 30° camera, using only three arms. The study involved 36 participants with varying experience levels who performed robotic tumor excisions on the models. The results demonstrated that experts performed significantly better than novices in terms of surgical margins, excision time, preserved parenchyma, and Global Evaluative Assessment of Robotic Skills (GEARS) scores. Expert group survey results showed an average score of 6.3/10 on realism of the model, 8.2/10 on the usefulness as training model and 6.9/10 score on the usefulness as an evaluation tool. The model was rated high for realism and training utility, confirming its face and content validity. The study concluded that the 3D-printed model was a valuable tool for training and assessing RAPN skills.

### 3.8. 3D printing simulator for vascular surgery

Alshomer et al. (27) explored the use of desktop 3D printing to create an affordable training tool for microvascular anastomosis. The model featured multiaxial and multiangular vessel orientations, designed with CAD software and printed using a thermoplastic polyurethane filament. Trials showed that the model provided realistic training scenarios, significantly aiding in the development of microsurgical skills. This approach represented a cost-effective and versatile method for enhancing microsurgical education.

The current 3D printing simulators for surgical training are listed in the following Tables 1–4.

**TABLE 1:** The list of the current 3D printing simulator for oral and otolaryngology surgery.

Study	Training objective	Materials and printing techniques	Advantage	Disadvantage	Cost
Seifert et al. (1)	Oral and maxillofacial surgery	Polylactic acid filament(PLA); fused filament 3D printer	More realistic with regard to the anatomical correctness, the degree of freedom of movement and the operative simulation	Poor results in the haptic feedback of the soft tissue	€3.46
Nica et al. (2)	Maxillary sinus augmentation	Laminated light-curing resins; digital 3D Printer	A Significant reduction of the surgery time of around 20%	–	–
Sonkaya and Bek Kürklü (3)	Direct and indirect pulp capping	Rigid photopolymer resin; multi-layer 3D-print	Perceived effectiveness of learning pulp capping and exposure recognition were evaluated as high-good	–	$0.60
Alrasheed et al. (4)	Ostiomeatal complex and frontal sinus for endoscopic sinus surgery	Vero white Plus; multi-material photopolymer printer	high visual appearance	–	–
Suzuki et al. (5)	Functional endoscopic sinus surgeries	–	Improved technical skills	–	–
Barber et al. (6)	Pediatric endoscopic ear surgery	Calcium sulfate hemihydrate powder; inkjet 3D-printing	Reduce surgery time	The lack of an external ear with anatomic features	$100
Chien et al. (7)	Otology surgery	Cast powder;-	Anatomically realistic	Lack of anatomic variations	*Phacon GmbH (Leipzig, Germany)
Frithioff et al. (8)	Mastoidectomy	layBrick filament;fused deposition modeling 3D printer	Improved technical skills	Insufficient for performing a safe mastoidectomy	$5
Iannella et al. (9)	Cortical mastoidectomy	Anycubic standard resin; photopolymers light-curing printer	Improved visualization; Hands-on learning; Customization; Reduced cost; Reproducibility;	Cost;Complexity;Time-consuming;Quality control;Material limitations;Limited haptic feedback;Environmental impact;Lack of standardization	€ 40
Nguyen et al. (10)	Augmented stapes fixation surgery	–	Versatility to compare the user friendliness of different techniques	Realism for ossicular chain palpation has not been objectively compared to a real ossicular chain	–
Takahashi et al. (11)	Temporal Bone Dissection	Plaster powder; full-color 3D printer	Tactile sensation of the model was excellent	The structure of stapes, tympanic sinus, and mastoid air cells were unsatisfactory	–
Narayanan et al. (12)	Endoscopic base of skull surgery	–	Suitable for learning registration, navigation and skull base drilling techniques	–	–

*Means commercial products.

**TABLE 2:** The list of the current 3D printing simulator for ophthalmology, neurosurgery and orthopedics surgery.

Study	Training objective	Materials and printing techniques	Advantage	Disadvantage	Cost
Famery et al. (13)	Descemet’s membrane endothelial keratoplasty	–	Feasible and valid teaching technique that permits to perform all but the descemtorrhexis step with a high success rate and a limited need of corneal material	–	–
Vatankhah et al. (14)	Orbital anatomy and pathology	–	Abstract reinforcement of points and content, stimulation of the imagination, enhancement of perception, increased student retention, expanded memory capacity	–	–
Nebor et al. (15)	Primary Dural Repair via an Endoscopic Endonasal Corridor	Polylactic acid; ultimaker 2+ 3D printer	Improved technical skills	–	$30
Uhl et al. (16)	Cranioplasty in Craniosynostosis	Biocompatible material; digital 3D printer	Easily replicated and customized based on patient-specific CT scan	Lack of the tactile feedback and sensation of performing surgery on actual patients.	–
Zhu et al. (17)	Endoscopic and Exoscopic Intracerebral Hematoma Surgery	–	good bone texture, the brain tissue texture and the experience in aspirating the hematoma	The surgical position, surgical approach and simulated brain tissue should be improved	$200
Zheng et al. (18)	Transnasal endoscopic surgery for pituitary adenoma	–; multimaterial 3D printer	Multimaterial and multicolor model for simulating individualized and complex anatomical structures in the sellar region	–	–
Encarnacion Ramirez et al. (19)	Endoscopic third ventriculostomy.	Resin; photopolymer printer	Cost-effective, reusable, and easily replicable	Lack of the texture of a fresh cadaver	$40
Byvaltsev et al. (20)	Microvascular training in neurosurgery	White polylactic acid materials; 3D printer with fused deposition modeling(FDM) technology	Appropriate hand position on the skull, becoming comfortable with the depth of the anastomosis, and simulating proper skull angle and rigid fixation	The absence of intracranial structures	–
Licci et al. (28)	Neuroendoscopic ventricular lesion removal	Polyvinyl alcohol; Replicator+ 3D printer	Low-cost, patient-specific, reusable	Mechanical proprieties to be improved	$94
Papavasiliou et al. (21)	Surgery for hand	Polylactic acid (PLA) filament; -	A great way forward for educating trainees that do not have access to courses or training, particularly in low resource settings	The trainees do not practice on dissecting the overlying tissue (raising Brunner flaps, protecting neurovascular bundle, tendon retrieval and pulley exposure)	–
Neijhoft et al. (22)	Surgery for the fractures of the ankle	–; Fused filament fabrication printing technology	Improved comprehension of complicated fractures	Lack of perception and haptic sensation of real bone	–
Ferràs-Tarragó et al. (29)	Arthroscopy surgery	Polylactic acid (PLA); fused filament fabrication (FFF) 3D printer	Accessible, economical, and effective.	–	$12

**TABLE 3:** The list of the current 3D printing simulator for gastrointestinal surgery.

Study	Training objective	Materials and printing techniques	Advantage	Disadvantage	Cost
Habti et al. (30)	Hand-Sewn Anastomosis of Small Bowel	Silicone; ultimaker 3D printer	Effective and inexpensive	Silicone texture to be improved	$35
Yang et al. (31)	Laparoscopic pancreaticojejunostomy	Silicone; dual-head silicone printer	Close to clinical reality	–	–
Xia et al. (32)	Laparoscopic intracorporeal intestinal anastomosis	Silicone; fused deposition modeling 3D printing	Excellent face, content, and construct validity	The intestine used in the experiment was single-layered, and it failed to simulate the actual structure of the human intestine	–
Xia et al. (33)	Laparoscopic choledochojejunostomy	Silicone; FDM 3D printer	Reduced surgery time	The current model does not contain blood vessels and simulation of intraoperative bleeding, the intestinal tract and bile duct used in this study were bilayer structures, lacking the multilayered structure of normal tissue	–
Holt et al. (34)	Endoscopic ampullectomy	silicone; -	Addresses the key requirements of a simulation model	–	$1,482

**TABLE 4:** The list of the current 3D printing simulator for urinary and vascular surgery.

Study	Training objective	Materials and printing techniques	Advantage	Disadvantage	Cost
Campi et al. (23)	Kidney Transplantation:	Silicone; -	Improving clinical outcomes and reducing the burden and cost of the learning curve	Simulation of ureterovesical anastomosis is not allowed	€ 5,000
Wong et al. (24)	Minimally invasive Urethrovesical anastomosis	Polymer; Lulzbot Taz 4 3D printer	Reduced learning curves	–	–
Reilly Scott et al. (25)	Partial nephrectomies	Polylactic acid (PLA); Ultimaker S5 3D printer	Inexpensive but detailed	High labor cost	$270–290
Hermans et al. (26)	Robot-assisted partial nephrectomy	Silicone; -	Specific designs, a lower cost and logistical ease	–	–
Alshomer et al. (27)	Microvascular Anastomosis	Thermoplastic polyurethane (TPU);Ultimaker 2 + 3D printer	Providing with multiaxial and multiangular vessel orientation during the anastomosis process	–	$1.30

## 4. Evaluation of the 3D printing simulator

### 4.1 Cost-effectiveness of the 3D printing simulator

In the studies, the cost of the 3D printing simulators seemed generally inexpensive. As mentioned by the studies, the lowest-cost 3D printing simulator was 3D-printed teeth with the cost of $0.60 and the most expensive simulator was 3D-Printed Training Model for Robot-assisted Kidney Transplantation with the cost of € 5,000. The average cost of the 3D printing simulators was between $30–300. However, as the studies described, there were extremely high potential labor cost during the 3D printing that hadn’t been calculated into. Frithioff et al. (8) mentioned that it took approximately 16 h to print a single temporal bone model, excluding time for maintenance of the printer. Iannella et al. (9) mentioned that printing and post-printing processes took 3 h and 1 h, respectively, for a total of 4 h required to produce each single model. Encarnacion Ramirez et al. (19) mentioned that for the time of segmentation, modeling in several iterations, and preparation of the prototype model for printing, they estimated approximately 12–25 h of working time. Reilly Scott et al. (25) mentioned that the average time their model took was 8 h to print (range 3–10 h), which was usually accomplished overnight. Licci et al. (28) mentioned that approximately 10–15 h of working time were estimated for the printing of the simulator. Therefore, although the direct cost of the 3D printing simulators was inexpensive, the overall indirect cost was hardly estimable which we should notice.

### 4.2 Limitations of the 3D printing simulator

The advantages of the 3D printing model were commonly acknowledged, including that customized, low-cost, patient-specific, reusable (16, 19, 25, 26, 28–30). However, in the current stage, the 3D printing simulator still have specific limitations. The most mentioned limitation was poor haptic feedback of the simulators (1, 9, 10, 16, 19, 22, 30), which was very important during the surgical training, since it is the key element for junior doctors to master practical procedures. Additionally, some simulators didn’t possess the integrated and elaborate structure as the human tissue, hence not the whole surgical procedures can be practiced by the simulators, and further improvement should be made (6, 8, 11, 17, 20, 21).

### 4.3 Surgical training curriculum with the use of 3D printing simulators

According to the review, we concluded some principles of 3D stimulator training curriculum. First, generalized simulators were usually used for medical students, aiming to give pre-clinical education and train their surgical skill. The customized simulators were usually used by surgeons for specific procedure training or pre-operative training. Second, subjective questionnaires were most commonly used to evaluate the generalized simulators by the medical students (1, 3, 7, 9, 10). However, there was no scale tool for the medical students to evaluate their understandings and surgical skills by the use of 3D stimulators. But, for the advanced and customized simulators, specific designed scale tools were usually used to evaluate the surgical capability of the surgeons, which can help them understand their weaknesses and improve their surgical skills better. Suzuki et al. (5) used the objective structured assessment of technical skills (OSATS) to evaluate the trainees’ performance in the functional endoscopic sinus surgery training curriculum by the use of 3D printing simulator. Licci et al. (28) used the global evaluative assessment of robotic skills (GEARS) and assessment of robotic console skills (ARCS) tools to evaluate the trainees’ performance in the robot-assisted kidney transplantation training curriculum by the use of 3D printing simulator. Campi et al. (23) used the modified anastomosis objective structured assessment of technical skills (MAOSATS) to evaluate the trainees’ performance in the laparoscopic intracorporeal intestinal anastomosis training curriculum by the use of 3D printing simulator. Hermans et al. (26) used the arthroscopic surgical skill evaluation tool (ASSET) to evaluate the trainees’ performance in the arthroscopy training curriculum by the use of 3D printing simulator. Besides, operative time was the most commonly used objective evaluation tool to evaluate the surgical capability of the surgeons (4, 6, 13, 15, 28, 32). In conclusion, generalized simulators can be used as teaching tools for medical students to have pre-clinical education and customized simulators can be used by surgeons for specific procedure training or pre-operative training. In the matter of assessments, specific scale tools and operative time are good methods to evaluate the surgical capability of the trainees in the 3D printing simulator curriculum, especially for the advanced and customized 3D printing surgical simulators.

## 5 Discussion

The application of 3D printing simulator in surgical training has been transformative, offering numerous benefits and opportunities for improving medical education and patient outcomes. 3D printing simulator can replicate the exact anatomical structures of patients, providing highly realistic models for surgical training. They offer a tangible and accurate representation of complex anatomies, which is crucial for understanding spatial relationships and practicing surgical techniques. Practicing on 3D printing simulator could reduce the risks associated with the first-time performance of new or intricate surgical procedures on actual patients.

Also, according to the cost-effectiveness, traditional surgical training methods, such as cadaveric dissection or live animal models, can be expensive and logistically challenging. 3D printing offers a more cost-effective and accessible alternative, with reusable and reproducible models.

Surgeons can use 3D printing simulator to plan and rehearse surgeries, leading to more precise and efficient procedures. Medical students and residents can practice basic and advanced surgical skills on 3D printing simulator, allowing for a safer and more effective learning curve.

However, there are specific barriers hindering the widespread adoption of 3D printed models in certain surgical fields. Some of the key barriers include: (1) Complexity of Anatomy: In certain surgical fields where the anatomy is highly complex or variable, creating accurate and detailed 3D printed models can be challenging. The intricate structures and variations in anatomy may require more advanced imaging techniques and expertise to generate precise models. (2) Cost and Resources: The cost associated with acquiring and maintaining 3D printing technology, as well as the resources required for creating high-quality models, can be a significant barrier. Not all medical institutions may have the budget or infrastructure to invest in 3D printing capabilities. (3) Training and Expertise: Utilizing 3D printed models effectively in surgical training requires training and expertise in both the creation of the models and their integration into educational curricula. Lack of training programs and expertise in this area can limit the adoption of 3D printed models in certain fields. (4) Standardization and Validation: The lack of standardized protocols for creating and validating 3D printed models for surgical training purposes can also be a barrier. Without established guidelines and quality control measures, there may be concerns about the accuracy and reliability of the models.

Addressing these barriers through increased investment in technology, training programs, and standardization efforts can help overcome the challenges and facilitate the wider integration of 3D printed models in all fields of surgical training.

## 6 Conclusion

Overall, 3D printing simulator have revolutionized surgical training, offering a realistic, customizable, and cost-effective method for preparing surgeons. As technology advances, their application will continue to expand, further enhancing the quality of surgical education and patient care.
